# Morphological and Structural Aspects of the Extremely Halophilic Archaeon *Haloquadratum walsbyi*

**DOI:** 10.1371/journal.pone.0018653

**Published:** 2011-04-29

**Authors:** Matilde Sublimi Saponetti, Fabrizio Bobba, Grazia Salerno, Alessandro Scarfato, Angela Corcelli, Annamaria Cucolo

**Affiliations:** 1 Department of Physics and Research Centre NanoMateS, University of Salerno and SPIN-CNR, Fisciano, Italy; 2 Department of Medical Biochemistry, Biology and Physics, University of Bari and IPCF-CNR, Bari, Italy; Université Paris Sud, France

## Abstract

Ultrathin square cell *Haloquadratum walsbyi* from the Archaea domain are the most abundant microorganisms in the hypersaline water of coastal salterns and continental salt lakes. In this work, we explore the cell surface of these microorganisms using amplitude-modulation atomic-force microscopy in nearly physiological conditions. We demonstrate the presence of a regular corrugation with a periodicity of 16–20 nm attributed to the surface layer (S-layer) protein lattice, striped domains asymmetrically distributed on the cell faces and peculiar bulges correlated with the presence of intracellular granules. Besides, subsequent images of cell evolution during the drying process indicate the presence of an external capsule that might correspond to the giant protein halomucin, predicted by the genome but never before observed by other microscopy studies.

## Introduction

**Figure 1:**
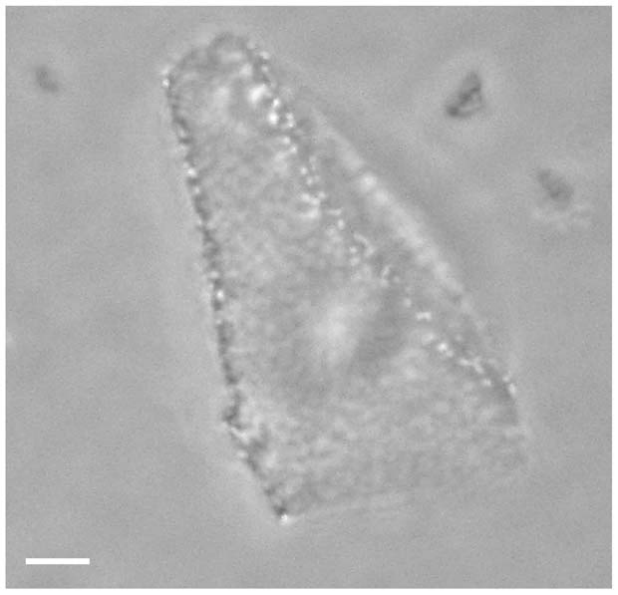
Optical phase-contrast microscopy image of a *H. walsbyi* square cell. The numerous light dots represent gas vesicles. Overall size is 10×10 µm. Scale bar 1 µm.

The extremely halophilic archaeon *Haloquadratum walsbyi* was first discovered in a coastal brine pool in the Sinai peninsula, Egypt, in the early 1980s; this novel organism aroused considerable interest in the scientific community because of its square cell shape and its ability to inhabit environments extremely rich in salts at the limit of water activity [1], [2]. For a long time, investigations into the physiology and biochemistry of *H. walsbyi* were very limited, because of the difficulties inherent in obtaining axenic cultures of the microorganism. Recently, two independent groups isolated and cultivated *H. walsbyi* and sequenced its genome [3], [4], [5]. Like other members of the *Halobacteriaceae* family, the *H. walsbyi* genome encodes photoactive retinal proteins of the membrane and S-layer glycoproteins of the cell wall [6]. One of the peculiarities arising from the genomic analysis is its ability to express the giant protein halomucin, which should play an important role in protecting cells against desiccation by creating an aqueous shield covering the cells. Recently, the lipidome of the square cells has also been described [7], [8]. To date, optical phase contrast, confocal fluorescence and electron microscopy analyses have been carried out to show the morphological features of these microorganisms, which are characterized in three ways: by a square or rectangular shape, typically measuring 2–5 µm wide by 0.1–0.2 µm thick; by the presence of highly refractive gas vesicles (Fig. 1); and by poly-β-hydroxyalkanoate (PHA) granules in the cytoplasm [1], [2]. Most microscopy studies have aimed to describe its intracellular organization, but only few details regarding the cell wall have been reported. Some authors describe a structural variability among different isolates. In one of the first studies on cells collected from natural samples, the square organisms showed a wall with a thickness ranging from 15 to 25 nm and with a regular structure consisting of circular particles. In freeze-fracture plane sections, it has also been observed that these particles appear to be arranged into orthogonal or hexagonal patterns with a spacing of approximately 23 nm; however, oblique sections suggest more complex patterns, which vary among the different microorganisms analysed [9], [10]. More recently, electron cryomicroscopy images of two different strains of *H. walsbyi*, C23 and HBSQ001, have revealed similar internal structures such as gas vesicles and poly-β-hydroxybutyrate (PHB) granules, whereas the cell wall of the isolate HBSQ001 showed an apparently three-layered complex structure [11].

In this work, we studied the haloarchaeon *H. walsbyi* strain HBSQ001 by Atomic Force Microscopy (AFM) to acquire new morphological details that had not yet been revealed by Electron Microscopy analysis. In comparison with previous high-resolution microscopy studies, by using amplitude modulation AFM, commonly referred to as either alternate contact (AC) mode or tapping mode, we analyzed native biological samples in nearly physiological conditions, thus minimizing any damage or artefacts due to fixation procedures [12], [13]. In addition, we exploited the unique capability of AFM to monitor in real-time the dynamic evolution of surfaces so as to better understand their morphological features and their changes during the variations in environmental conditions. In this study, two different AFM experiments were carried out: in the first, we explored the cell surface in an attempt to identify areas with different elasticity properties, while in the second, by continuously imaging cells during the drying process, we demonstrated the presence of a capsule appearing as a thin film external to the cell wall that, in archaea, is constituted only by the surface layer (S-layer).

## Materials and Methods

### Microorganism culture

We studied the haloarchaeal cell *H. walsbyi* (strain HBSQ001) provided by F. Rodríguez-Valera. The square cells were grown in liquid growth medium (HAS medium) containing per liter: 195 g NaCl; 50 g MgSO_4_·7H_2_O; 35 g MgCl_2_·6 H_2_O; 5 g KCl; 0.25 g NaHCO_3_; 1 g NaNO_3_; 0.5 g CaCl_2_·2 H_2_O; 0.05 g KH_2_PO_4_; 0.03 g NH_4_Cl and 20 ml Tris-HCl (1 M, pH 7.4), supplemented with 0.5 g glycerol, 0.1 g yeast extract and 1 g sodium pyruvate. The strain was grown aerobically in light in a gyratory shaker at 80 rpm and 40°C [3].

### AFM analysis

We operated both on dried and on liquid samples. Liquid samples were prepared by applying a 10 µl droplet of cells in the stationary growth phase onto freshly cleaved mica and removing the excess liquid by pipetting back the upper part of the deposited drop solution. Dried samples were prepared by applying a droplet of cells in the stationary growth phase on mica for 30 minutes followed by rinsing with ultrapure water and by drying with N_2_. Samples were immediately transferred into the AFM microscope and were imaged by AFM in AC mode using a Multimode Nanoscope V system (VEECO, Santa Barbara, CA) operating both in liquid and in dry conditions. The microscope was equipped with a 15 µm scanner (EVLR-scanner) and a liquid cell with no o-ring seal was used. During the experiments, the laboratory room temperature was kept constant with a variation of less than 0.3°C to minimize thermal drift effects.

In AC mode, a stiff cantilever is oscillated by means of a sinusoidal driving force using a piezoelectric crystal. When close to the surface, the tip of the cantilever intermittently taps the sample at or near the cantilever's resonant frequency. The cantilever oscillation is necessarily reduced due to energy loss caused by the tip touching the surface, and the reduction in oscillation amplitude is used to identify and measure surface features. By scanning this tapping cantilever over a selected area of the sample, the cantilever oscillation amplitude is maintained constant by a feedback loop that modifies the average tip-surface separation. This provides a measurement of the surface corrugation, affording height profiles for each line, in other words imaging the surface topography.

Simultaneously, the phase shift between the cantilever oscillation and the oscillating driving force can be measured by means of phase imaging. In this case, as consequence of tip/sample interaction, different material properties of the surface are correlated with different phase shifts, and so phase imaging can be used to identify areas from a sample in terms of such differing properties as stiffness, adhesion, and viscoelasticity. The optimal oscillation frequency is different for height imaging and for phase imaging. In the former, it is convenient to operate at an oscillation driving frequency lower than the cantilever resonance frequency so that the gain can be calculated from the higher slope of the amplitude frequency response curve. By contrast, in order to optimise phase imaging, it is convenient to operate with a driving oscillation at the cantilever resonance frequency so as to get gain from the higher and more linear slope of the phase-frequency response curve at the expense of reduced topographic height resolution. Finally, when optimising for phase imaging and/or when the feedback amplitude is not correctly regulated, images obtained by acquiring the amplitude error signal, commonly referred to as amplitude imaging, also revealed topographic features with high lateral resolution but without any three-dimensionality.

Operating in AC mode on soft biological samples eliminates lateral forces such as friction, adhesion, and electrostatic dragging, thus improving lateral resolution and allowing imaging for specimens poorly adsorbed on a substrate.

**Figure 2:**
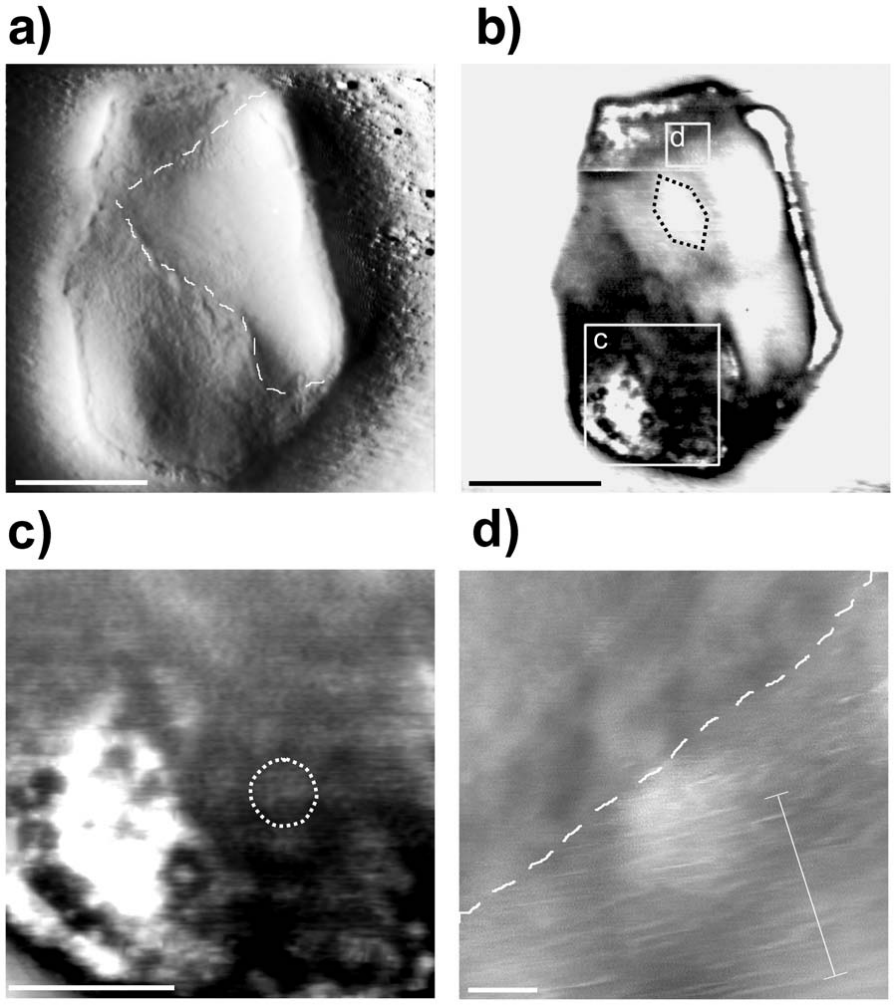
AFM images of a *H. walsbyi* folded cell. Amplitude (a) and phase (b) images show a cell with its upper right-hand corner folded. Enlarged images of the white boxed areas show the spotted region (c) and the striped region (d); the dashed line is a guideline along the border of the folded corner. Scale bars: 1 µm (a, b), 400 nm (c), 100 nm (d).

**Figure 3:**
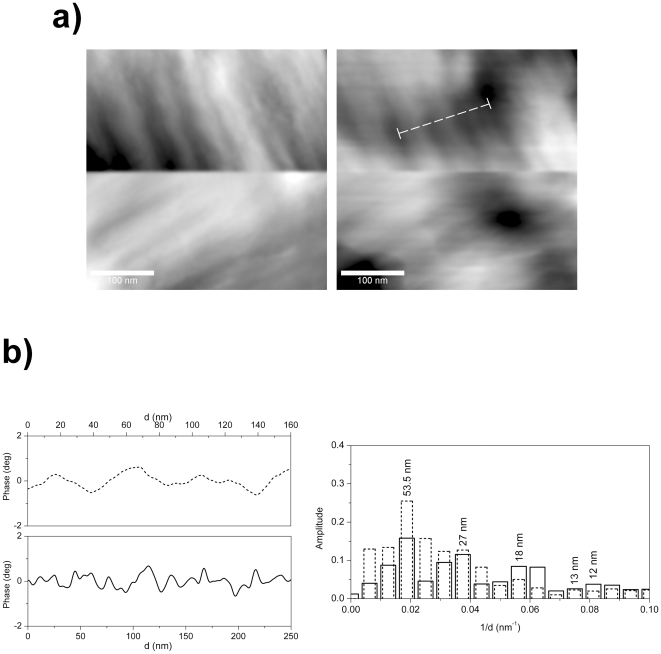
AFM analysis of striped regions. a) Amplitude and phase images of striped regions observed on a dried cell. b) Average profile line extracted from the region indicated with the dotted line in the phase image and its Fourier analysis results in the bar graph. The same analysis was applied to the striped region indicated with the full line in Fig. 2d.

**Figure 4:**
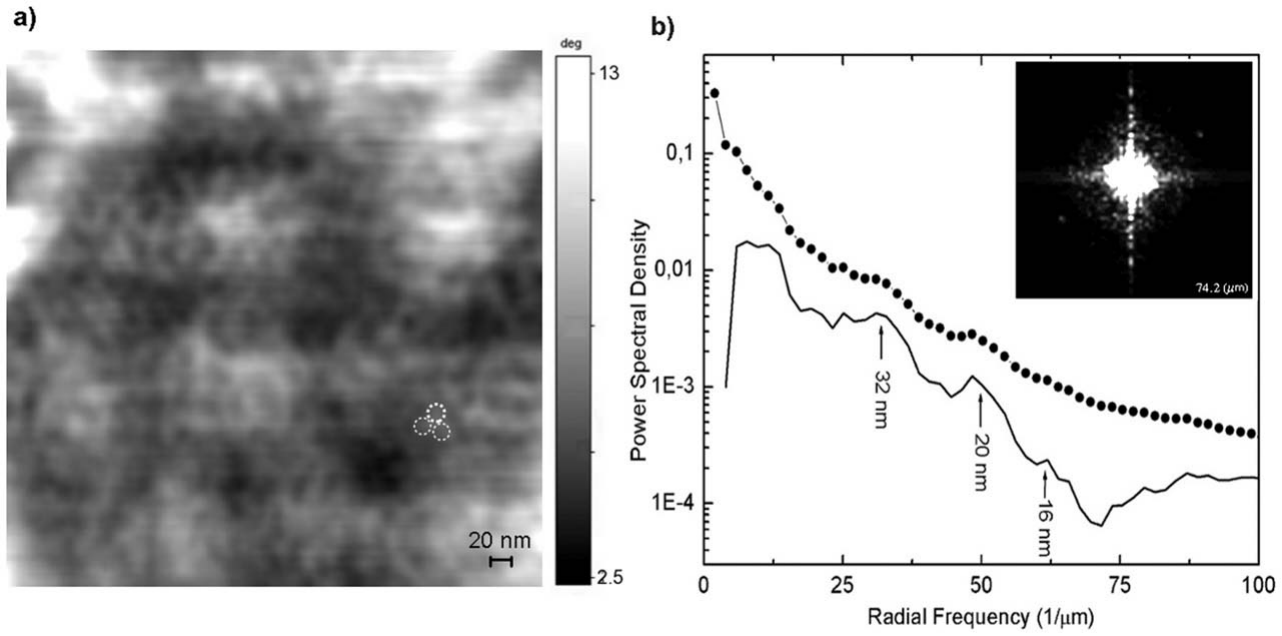
AFM analysis of *H. walsbyi* S-layer. a) Zoom view of Fig. 2c, showing the S-layer corrugation made up of units 16–20 nm apart represented by dashed circles. Scale bar is 20 nm. b) Fourier analysis. In the inset is the 2D PSD image within a frame of ±74.2 µm^−1^. Average radial profile of PSD versus spatial frequency (dotted line) and its enhancement after the subtraction of the 1/f background (full line).

**Figure 5:**
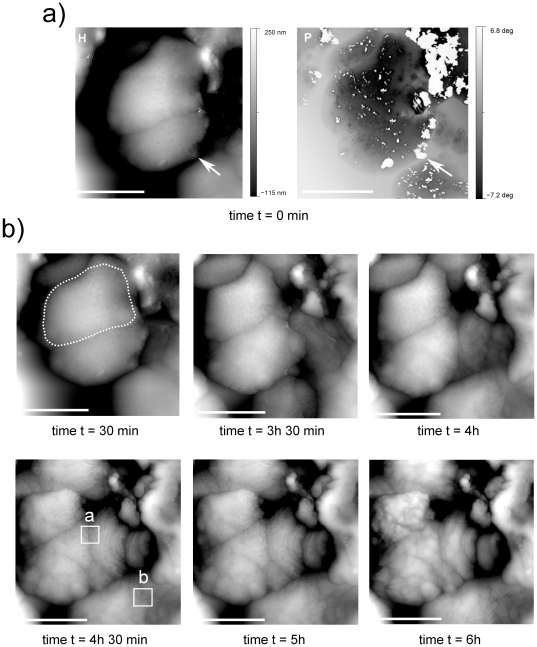
AFM images of *H. walsbyi* cell evolution during the drying process. a) Height (H) and phase (P) images of cells at the beginning of the process (time t = 0). In height imaging, cells present an external capsule appearing as a thin film disrupted in some locations. One big laceration is indicated by the white arrow. In phase imaging, capsule lacerations correspond to white spots. In the following height images (b) acquired at different times during the drying process, the cells decrease in volume and lose their capsule, uncovering the underlying cell layer. Scale bar 2 µm.

Effectively, in our experiments, after the tip was engaged on a selected point of the surface, the scan range was gradually increased, while optimizing appropriate scan parameters for the sample's roughness. The rule used to get low noise and low applied tip forces was to identify a minimal driving amplitude and maximal tapping amplitude setpoint. At low magnification (frame size of about 3–5 µm), imaging was performed with scan rates in the range of 0.3–1 Hz while, at high magnification (sub-micron frame size), scan rates were kept in the 1–3 Hz range. Images were recorded simultaneously in both height, amplitude error and phase modalities. In height imaging, the driving frequency was lower than the resonance frequency so as to get higher sensitivity in the tapping amplitude variations, whereas when optimizing for phase shift imaging the operating frequency was kept very close to the resonance. In height imaging, integral and proportional feedback gain parameters were set as high as possible to faster react to changes in topography and just below the onset of spurious feedback oscillations. Finally, the Z-scan range parameter was set to the minimal appropriate value for the surface roughness in order to maximize the instrumental resolution along the Z-axis. The experiments reported in Figs. 2, 3 were performed by using antimony (n)-doped Si cantilevers with an elastic constant of 48 N/m, a resonance frequency of 188 KHz and a nominal tip radius of 10 nm (NCLV VEECO), while the results in Figs. 4, 5 were obtained by using super sharp antimony (n)-doped Si cantilevers with 42 N/m, 320 kHz and a nominal tip radius of 2–5 nm (TESP-SS VEECO).

## Results

When the archaeon *H. walsbyi* is observed in the optical phase-contrast microscope, it appears as a square or rectangular sheet with light dots representing intracellular refractive gas vesicles. Often, larger cells do not lie flat on the glass slides, but rather fold one or more corners so that they look irregularly-shaped rather than rectangular or squared. For example Fig. 1 presents a square cell with its right-hand side folded.

In our study to image *H. walsbyi* cells by AFM, we operated both in air and in liquid conditions. When operating in air with dried samples, NaCl salt crystals were removed by rinsing with high purity water after a drop of suspension had deposited on the substrate and an appropriate time had passed. Nevertheless, during the rewashing procedure, most of the cells, especially the biggest ones, were lost or detached from the substrate. On the other hand, imaging in liquid involves problems such as low cell adhesion to the substrate.

Since operating on dried cells does not completely fulfill the requirement of imaging in physiological conditions, we experimented with several different methods in order to allow liquid imaging. By using solutions at high cell concentrations, we reduced cell mobility and maximized rapid identification of cells for imaging purposes; however, this also entailed the disadvantage of cell agglomeration, which of course reduced our chances of imaging one single flat cell at a time. In liquid conditions, we operated on a thin film of microorganisms suspension, thick enough to keep the *H. walsbyi* cells humid for a period of some hours as estimated by our AFM results. This provided a good compromise for liquid measurements, as it enabled the imaging to start under physiological conditions. Various substrates were tried out during our study: freshly cleaved mica, glass, silica, polystyrene, agarose, special etched grid substrates with micrometer pitch matching typical *H. walsbyi* cell size and substrates treated with poly-lysine were all tested [14], [15], [16], [17]. In our experience, none of the latter methods proved to be particularly efficient, with the best results being obtained with a freshly cleaved mica surface.

Fig. 2 presents AFM images of a single cell acquired in liquid conditions with a super sharp tip and a point/line data acquisition resolution of 4.7 nm. Fig. 2a and 2b are respectively amplitude and phase images of the whole cell, while Fig. 2c and 2d are enlargements of different regions of the phase image. Amplitude imaging is an imaging mode that displays topographical features at the sample surface, while phase imaging is an imaging mode which goes beyond topographical data to detect variations in composition, adhesion, hardness and other properties of the sample [18]. In the amplitude image (Fig. 2a), the cell appears as a large square (∼3 µm) with its upper right-hand side folded. The cell is lying flat on the mica substrate, thus allowing a detailed analysis of the cell S-layer which shows a granular corrugation. Interestingly, in the phase image (Fig. 2b), the surface appears characterized by a spotted corrugation formed by a quite regular, closely-packed arrangement of rounded domains with an average size of about 100 nm (dashed line in Fig. 2c). These white circular regions are not clearly visible in the amplitude image, but are observed in the phase image, leading us to hypothesise that they are related to variations in the elastic response to the tapping cantilever. In other words, this indicates that when imaging the *H. walsbyi* surface in tapping mode, we are probably dealing with a soft surface moulding itself over stiffer internal bodies, thus allowing for inspection of harder rounded intracellular vesicles or granules.

Looking at the white regions located at the right-hand folded corner as well as at the lower left corner, the presence of gas vesicles can also be discerned (see dashed line in Fig. 2b). They can be recognized by shapes looking like the well-defined cylinders closed by conical end caps mentioned in ref. [11]. Gas vesicles confer buoyancy and therefore aid cells to position themselves close and parallel to the water surface, thus optimizing light absorption by the photoactive retinal proteins. Moreover, interestingly, the folded side of the cell, at the top right-hand corner, appears characterized by an additional striped corrugation (Fig. 2d). Such peculiar striped corrugations were also observed on other samples. For example, Fig. 3a reports amplitude and phase images of a striped region measured on the surface of a dried cell (Fig. S2). The scan direction was rotated by 90° once half of the image had been completed: the stripes followed the rotation, thus indicating that these stripes were not an artefact due to the tip or to some external vibrations or self oscillation. The result of varying scan parameters such as scan rate and feedback loop gains, in the range of reasonable conditions, produces a negligible effect on the phase image. To highlight the presence of a striped periodic structure on the cell surface and to better quantify the periodic lengths, Fourier analysis was used [19]. An average curve was first extracted from 36 adjacent profile lines, in the region indicated in Fig. 3a with dotted lines, and its Fourier spectral weights were calculated. Results, reported in the bar graph of Fig. 3b (dotted line) together with the corresponding averaged profile line show that striped structures yield higher peaks in the Fourier spectrum standing out from the morphological background. A high peak was observed at 53.5 nm corresponding to the clearly visible wide stripes in the phase image, and other lower peaks at 27 nm, 18 nm and 13 nm corresponding to periodic structures that are hard to see without zooming in.

By applying the same average procedure of profiles and subsequent Fourier analysis to other striped regions, observed on different *H. walsbyi* cells images and across different AFM experiments, we get similar Fourier Spectra with peaks at the same characteristic length values. For example in Fig. 3b full lines show results obtained from the striped region of the folded cell described above (Fig. 2d). In the bar graph, we found a high peak at 53.5 nm and other maxima at 27, 18 and 12 nm.

Going back to the cell reported in Fig. 2, higher magnification of the spotted region reveals details of the S-layer (Fig. 4a). As well as the big white spots described above, the presence of regular small scale corrugations can be observed. To better quantify the spatial frequency content, Fourier analysis was used. In the inset of Fig. 4b, the image of the power spectral density (PSD) is presented showing a significant peak, clearly visible in the first-third quadrant, at the spatial frequency of 50 µm^−1^ corresponding to a period of 20 nm. The respective average radial profile (dotted line Fig. 4b) versus the spatial frequency *f* was then calculated showing peaks at 32 nm and between 20 nm and 16 nm. This excess of harmonic components can be enhanced by subtracting the 1/f dominantly low frequency content from the average radial profile (full line Fig. 4b) and for this purpose the radial amplitude of the Fourier transform was fitted to k*1/f, with k = 2.17 (regression coefficient = 0.98). The spectral analysis is indicative of the presence of a pseudo-periodic corrugation, embedded in the surface morphology, with a defined period of 20 nm and a spreading of spectral components around 16 nm and 32 nm. This corrugation is in agreement with calculated data for S-layer hexagonal units in archaeal square microorganisms [20].

**Figure 6:**
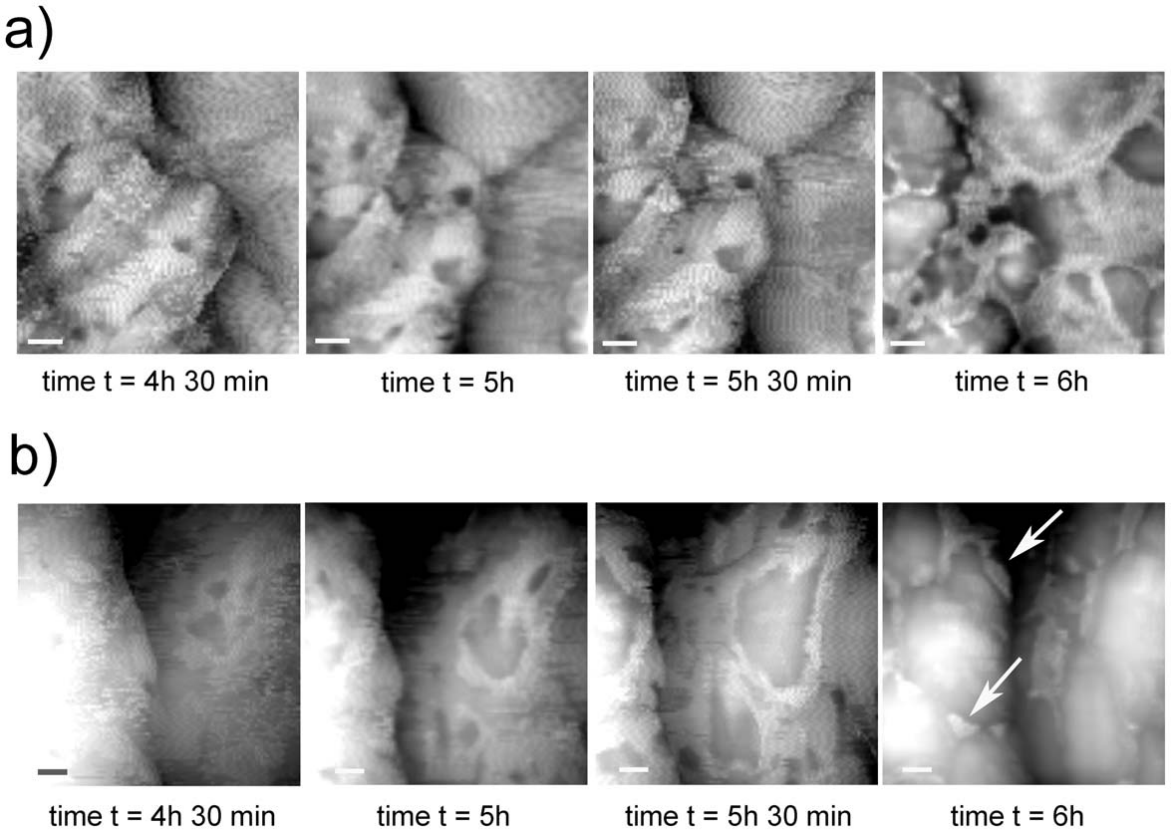
AFM images of *H. walsbyi* external capsule disruption during the drying process. Height images are enlargements of two representative regions (white boxes in Fig. 5b time t = 4 h 30 min) showing capsule evolution during the drying process. The capsule features holes or lacerations that break up over time, gradually increasing in size. At the end of the process remnants of the capsule can still be seen, mainly located in the valleys between the protrusions (white arrows time t = 6 h). Scale bar 100 nm.

In Figs. 5 and 6, we present a second experiment in which we studied the changes to the topography of *H. walsbyi* cells during the drying process, from liquid to completely dried-out. To follow the process at a rate of one image per half hour, a line scan rate of 0.3 HZ with a 512 point-line sampling was used, resulting in a data acquisition resolution of 9.5 nm, striking a good compromise between imaging quality, experimental stability and time intervals.

Fig. 5 presents a selection of images during the drying process. In this case, images show cell agglomeration probably arranged into piles. In these experimental conditions, the peculiar square shape of these microorganisms is clearly less evident, but can be identified by the dashed line that makes the border of one of the cells more visible (Fig. 5b t = 30 min).

At the beginning of the process (t = 0), in liquid conditions, the cell surface is smooth (Fig. 5a H), showing up dark in the contrast of the AFM phase image (Fig. 5a P), indicating soft material. The smooth surface seems to be disrupted or ripped in some locations, especially along the borders. These lacerations correspond to the white contrast patches seen in the AFM phase image, indicating dry or stiffer material. In particular, the big laceration indicated by the white arrow in the figures appears to be due to a sharp protuberance of some inner parts of the cell. This observation suggests that we are dealing with a thin capsule surrounding the cell body, as predicted by the genome and described in literature as a structure essential for forming an aqueous shield. [6]

The next six images (Fig. 5b) were acquired at different times during the drying process. Note that the second image in the series was taken three hours after the first, while the others follow every thirty minutes. At the beginning, some of the existing lacerations show a tendency to be dynamically repaired. Later, cells seem to lose their outermost capsule which over time is disrupted at several locations, thus uncovering an underlying cell layer. This is confirmed by the corresponding AFM phase images that show a gradual increase in white zones, with a complete change from dark to white by the end of the desiccation process thirteen hours later (not shown here for the sake of brevity). Besides, during the process the cell volumes decreased, which seems to be related to water loss affecting different cells to different degrees. When the outermost capsule is almost completely torn, about six hours from the start, the final observed corrugation mainly shows the surface of the underlying layer, which appears gibbous probably due either to the presence of harder intracellular vesicles or granules which become more evident once the cell is dry, or to the destruction of this layer due to the drying process (Fig. S1). Cells with a lower volume also show smaller surface protrusions.

Fig. 6 shows two enlargements of Fig. 5b time t = 4 h 30 min in random representative regions. Here, the disruption of the external capsule features holes or peculiar-shaped lacerations that break up over time, gradually increasing in size and then suddenly collapsing. Even though some surface modification due to the tip scan activity can be observed, seen in sporadic horizontal prolongations of the fibers accordingly with the horizontal tip scan movements, the main cause of the capsule disruption seems to be water loss. Still, at the end of the experiment, even the complete dried samples showed some remnants of the capsule, mainly located in the valleys between the protrusions (white arrows in Fig. 6b t = 6 h).

## Discussion

In this work, the AFM tapping mode technique was used to study the archaeon *H. walsbyi*. Although several biochemical features of *H. walsbyi* have been described in previous studies, few microscopic studies and observations of the cell surface topography have been reported. Our images were obtained operating in liquid under physiological conditions. On imaging the cellular surface, we observed a spotted corrugation due to the presence of hard rounded intracellular bodies which we can attribute to PHB granules (Fig. S3). These granules appear almost all the same size inside a single cell and tightly packaged in a bag. They are known to act as carbon and energy storage compounds but, from a physiological point of view, they could also serve to reduce the cytosol volume, decreasing the cellular energy demand for ionic homeostasis. They are accumulated in unbalanced growth conditions i.e. in conditions of nitrogen limitation and abundant carbon sources, and are reutilized when the carbon sources gets exhausted. The chemical and physical properties of PHB are similar to synthetic or petrochemical-based plastics such as polypropylene, polyurethane, vinyl chloride and hexachloroethane. It can therefore be used as biodegradable plastic for waste management strategies. In square cells PHB granules are usually abundant,which is interesting if we consider that nowadays the use of PHB is limited by its high industrial production costs [21].

When imaging the cellular surface, we sometimes also discerned the presence of striped corrugations which do not appear to be homogeneously distributed but rather localized in a few regions. They can be seen on the folded side of the cell in Fig. 2 while in experiments on completely dried samples they are near the cell borders. It is not clear whether they correspond to external or internal structures and further experiments are needed to clarify their nature. In other microorganisms, striped domains have been observed in gas vesicle membranes [22], [23], in the peptidoglycan cell wall of some bacteria [24] and as protein-lipid domains in cellular membranes [25].

With regard to the S-layer, this is the outermost cell envelope commonly found in bacteria and archaea. It consists of identical protein or glycoprotein subunits arranged in a crystalline lattice. In many archaea it is the only cell wall component and therefore provides not only a chemical and physical protection but also a mechanical stabilizing function. In *H. walsbyi*, in order to obtain good images of the S-layer surface structure, higher resolution experiments on a few nanometer scale are needed; nevertheless, in Fig. 4 a fairly regular corrugation with a period of 16–20 nm can be measured across the whole cell surface, in good agreement with the value reported in literature for the lattice constant of the square microorganism S-layer [20]. Besides, in experiments on samples dried for more than 12 hours, we often observed, in locations close to cells, the formation of reassembled patches with the same distinctive pattern on the substrate surfaces. Such patches with a 16 nm, nearly hexagonal lattice and with a height of 10 nm are formed on the silica as well as on glass and mica substrates. This 2D self assembling reconstruction process is one of the well known peculiarities of S-layer proteins which has recently been used to realize matrices for the binding of functional molecules such as enzymes, antibodies, antigens etc [26].

Unlike many archaea, in *H. walsbyi* the S-layer is not the only cell wall component. In this study by following the cell surface evolution during the drying process, we discerned the presence of an outermost cell layer, an external capsule, which could be attributed to the external halomucin capsule (Fig. S3). Halomucin is an extremely large protein, homologous to the mammalian mucins, which play an important role in protecting tissues against desiccation processes. AFM imaging of animal mucins visualizes long filaments [27] which resemble the strands observed in the images presented here, acquired during the drying process (Fig. 6). Besides, as can be seen in Fig. 5, at the beginning the capsule appears to be swollen with water, collapsing later during the drying process, and this observation confirms the idea of a capsule acting as a water reservoir to avoid desiccation and to regulate water activity. Alongside this function, it has been suggested that halomucin might establish the framework of a cross-linked extracellular matrix contributing to the rigidity and maintenance of *H walsbyi*'s unique square cell morphology [6]. At the same time amino acid sequence analyses suggest partial homology to cellular adhesion molecules and degradation enzymatic activity, electing halomucin responsible for spatial arrangements of *H. walsbyi* cells in the form of postage stamp sheets and for the utilization of the scarce resources of the environment (http://www.mosaic.ethz.ch/research/docs/Rinck2009.pdf).

A deeper knowledge of both biological functions and physical and chemical properties of halomucin should provide insights for biomedicine, pharmacology and biotechnology. The structural and functional similarity to mammalian mucins should give us an idea of the potential importance of halomucin. For example it could be used to create engineered biological filters for microorganisms and unwanted molecules, to improve drug delivery effectiveness and as polymeric gel for biosensors or other biotechnological devices [28]. AFM tapping was successful with regard to the difficulties of imaging this delicate biological capsule which had until this work not yet been imaged.

## Supporting Information

Figure S1 **AFM images of** ***H. walsbyi*** **dried cells.** Amplitude error, phase and height images with the corresponding profile lines extracted from the regions indicated by the white lines. Cells appear to be made up of intracellular vesicles. The cell on the left has been defoliated of an external sheath which is still attached to the left-hand side of the cell. This effect may be due either to the drying or to the mechanical effect of the tapping AFM tip during the first fast recognition scan over a very large area (70 µm).
(TIF)

Figure S2 **AFM images of a** ***H. walsbyi*** **dried cell damaged on its external surface.** Amplitude error, phase, height and profile lines images at time t = 0 (a) and after 24 hours (b). At time t = 0 profile line extroflections corresponding to the halftone gray zones in the phase image can be interpreted as the external surface envelope while the sharp dips and depression areas, corresponding to zones shaded light gray and black in the phase image, are assumed to be scratches that uncover intracellular materials such as gas vesicles or PHB-granules and cytoplasm. Looking in detail amplitude and phase images it can be observed that most of the internal regions show a striped texture while the external areas, i.e. the undamaged regions, show a spotted texture. After 24 hours (b) the black areas previously found on the cell borders (a) were disappeared, as to be representative for a liquid material completely dry, and the external spotted envelope is clearly less extended than before, having uncovered some intracellular vesicles.
(TIF)

Figure S3 **Fluorescence microscopy image of** ***H. walsbyi*** **cells.** In red PHB granules stained by Nile blue, in green halomucin stained by a specific antibody coupled to fluorescein (unpublished data available on line at http://wwwmosi.informatik.uni-rostock.de/cmsb08/pdfs/talk_oesterheldt.pdf. Courtesy of Prof. Dieter Oesterhelt, Max Planck Institute of Biochemistry, Munich, Germany. e-mail: oesterhe@biochem.mpg.de).
(TIF)
