# Supplementary figures and images for: Morphological and Structural Aspects of the Extremely Halophilic Archaeon Haloquadratum walsbyi
Source: PLoS One. 2011 Apr 29;6(4):e18653. doi: 10.1371/journal.pone.0018653 (PMC3084702; doi:10.1371/journal.pone.0018653)

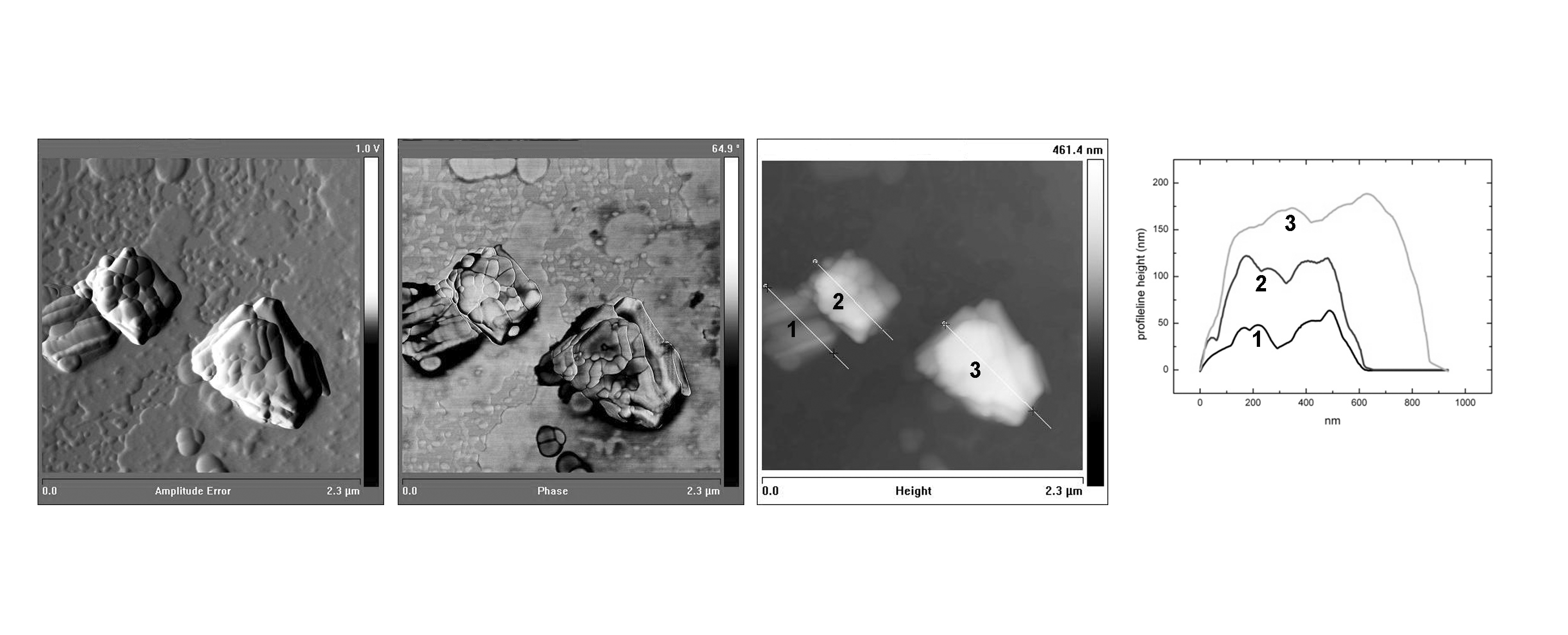

Supplement: Figure S1 — AFM images of H. walsbyi dried cells. Amplitude error, phase and height images with the corresponding profile lines extracted from the regions indicated by the white lines. Cells appear to be made up of intracellular vesicles. The cell on the left has been defoliated of an external sheath which is still attached to the left-hand side of the cell. This effect may be due either to the drying or to the mechanical effect of the tapping AFM tip during the first fast recognition scan over a very large area (70 µm). (TIF) [file pone.0018653.s001.tif]

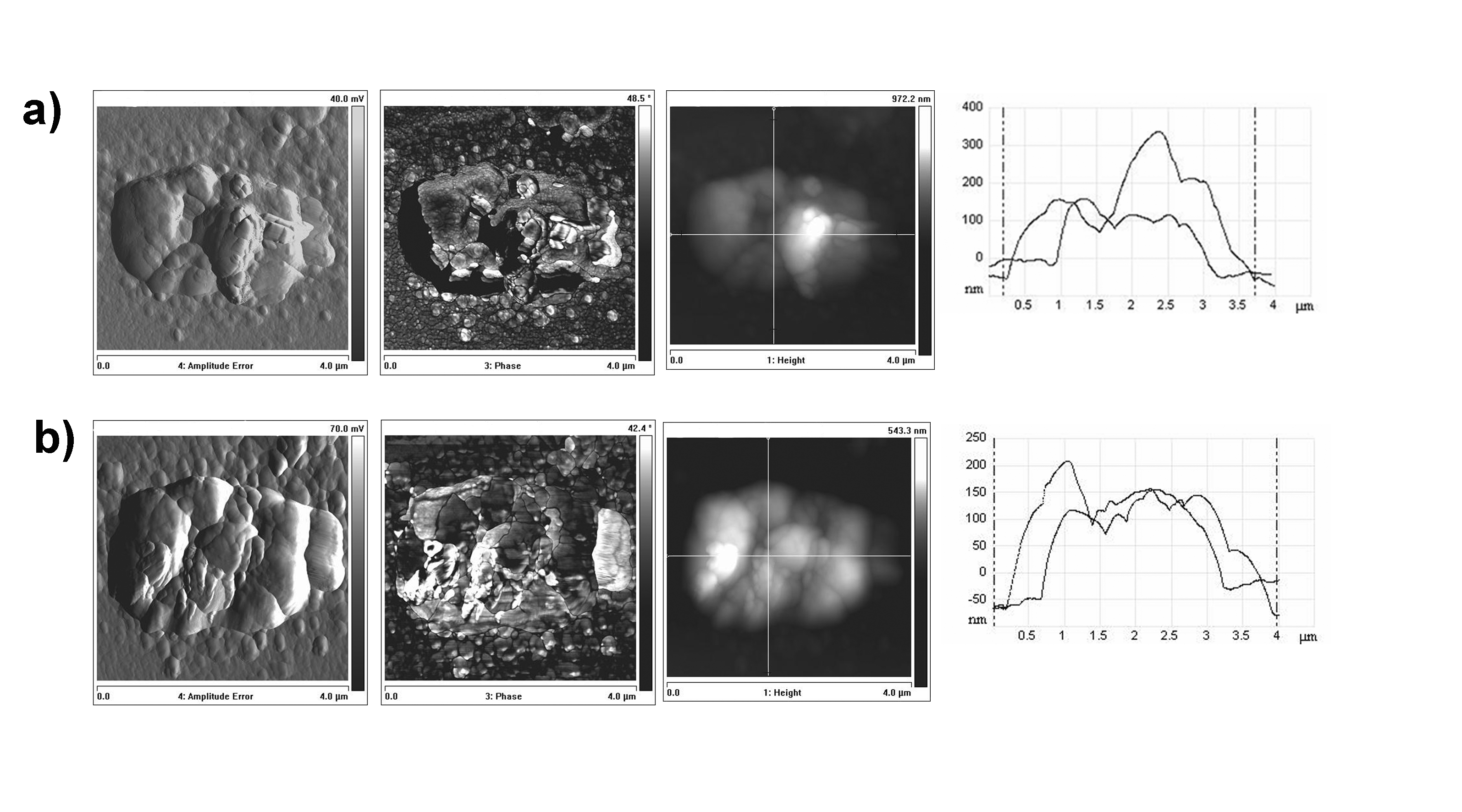

Supplement: Figure S2 — AFM images of a H. walsbyi dried cell damaged on its external surface. Amplitude error, phase, height and profile lines images at time t = 0 (a) and after 24 hours (b). At time t = 0 profile line extroflections corresponding to the halftone gray zones in the phase image can be interpreted as the external surface envelope while the sharp dips and depression areas, corresponding to zones shaded light gray and black in the phase image, are assumed to be scratches that uncover intracellular materials such as gas vesicles or PHB-granules and cytoplasm. Looking in detail amplitude and phase images it can be observed that most of the internal regions show a striped texture while the external areas, i.e. the undamaged regions, show a spotted texture. After 24 hours (b) the black areas previously found on the cell borders (a) were disappeared, as to be representative for a liquid material completely dry, and the external spotted envelope is clearly less extended than before, having uncovered some intracellular vesicles. (TIF) [file pone.0018653.s002.tif]

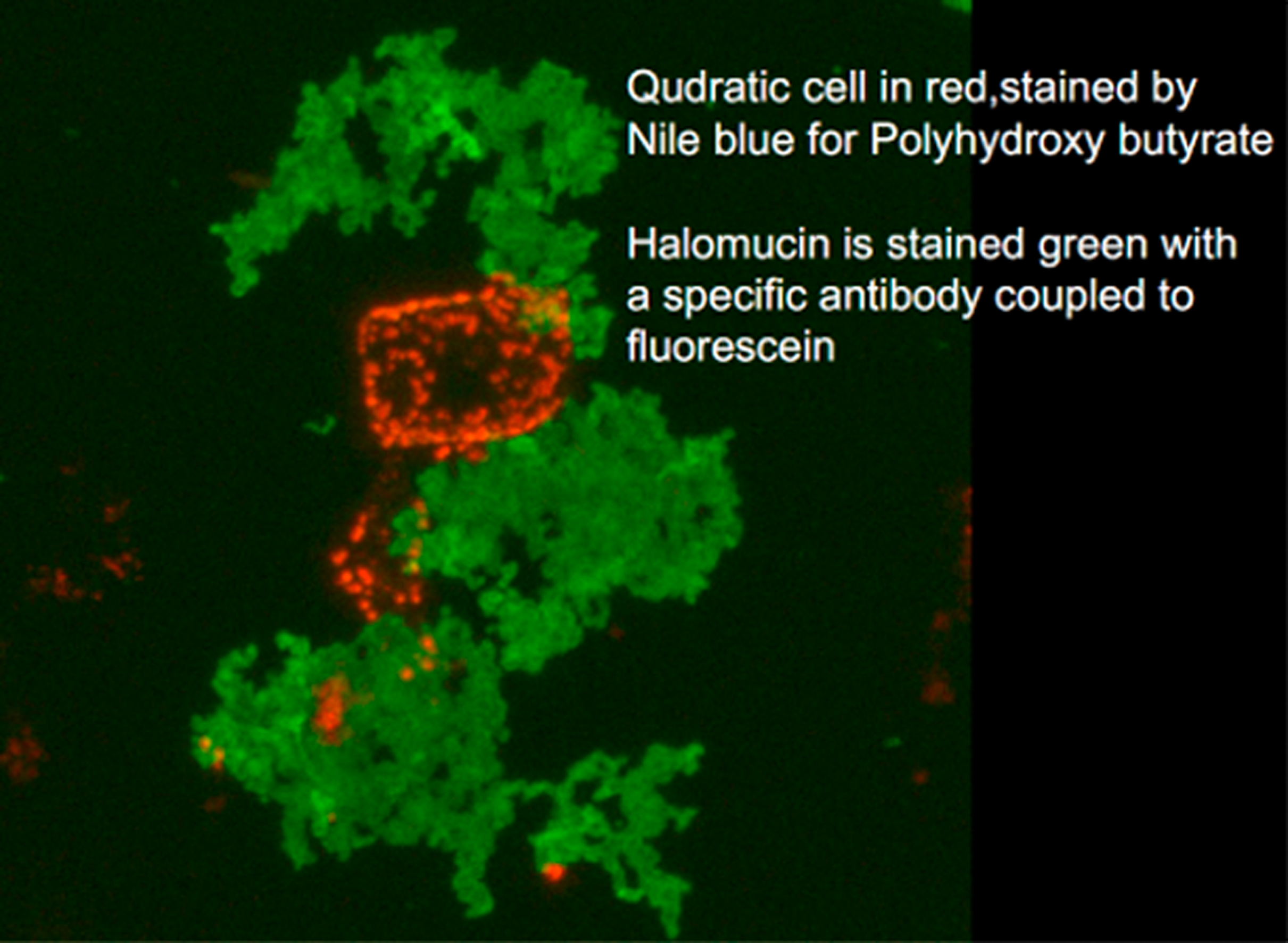

Supplement: Figure S3 — Fluorescence microscopy image of H. walsbyi cells. In red PHB granules stained by Nile blue, in green halomucin stained by a specific antibody coupled to fluorescein (unpublished data available on line at http://wwwmosi.informatik.uni-rostock.de/cmsb08/pdfs/talk_oesterheldt.pdf. Courtesy of Prof. Dieter Oesterhelt, Max Planck Institute of Biochemistry, Munich, Germany. e-mail: oesterhe@biochem.mpg.de). (TIF) [file pone.0018653.s003.tif]
